# Ligation of the Dorsal Vein of the Penis for Impotency

**Published:** 1897-12

**Authors:** Everet E Tracy

**Affiliations:** 1003 Columbus Memorial Building; Chicago


### LIGATION OF THE DORSAL VEIN OF THE PENIS FOR IMPOTENCY.

BY EVERET E TRACY, M. D., CHICAGO.

For years impotency has been treated by suggestion, correction of nervous reflexes and stimulation of the organs of generation by all the aphrodisiacs known. Corrections of the blood supply have, however, always been overlooked.

For the proper understanding of the treatment to be described, a brief reference to the anatomy is neccessary. The penis is an organ controlled partially by the nervous and partially by the circulatory system. More than two-thirds of the bulk of the penis is formed by the corpora cavernosa, which are separated from each other by a septum from which fibro-elastic bands diverge in all directions to form trabeculæ. These are filled by blood-vessels to form erectile tissue, and receive their blood supply from the arteries of the corpora cavernosa, branches of the dorsal artery of the penis, from the internal public.

Under the corpora cavernosa lies the corpus spongiosum, which commences as a bulb below the crura and in front of the triangular ligament, expanding anteriorly into the glans penis. This receives its blood supply from the artery of the bulb. The arteries of all three bodies finally terminate in erectile tissue spaces. From these spaces the veins begin. Some wind around the side of the organ to the dorsal vein, while others pass under the pubes to the prostatic plexus.

During an erection the blood leaves the penis by two channels, the dorsal vein and the prostatic plexus. Should there be any enlargement of the dorsal vein or should the blood be carried off too rapidly by the prostatic plexus, it would leave the organ faster than the arteries could furnish it.

In order to overcome this abnormal condition it is necessary to retard the flow of venous blood from this organ by cutting off the return flow by the dorsal vein. The diagnosis of impotency from enlargement of the dorsal vein, or too rapid emptying of blood from the penis, is easily made. In impotency from the lack of development of the spinal center we find well-developed genital organs, but absence of sexual desire and power of erection. Impotence due to exhaustion of the cortical center manifests itself by an immediate erection and discharge, without any feeling of gratification. In impotency from enlargement of the dorsal vein the sexual appetite is usually present, but the patient fails to obtain an erection, or has only a semi-erection. The dorsal vein appears large and varicosed. In one case I observed a dorsal vein on either side of the penis.

To confirm the diagnosis I have adopted the plan suggested by Dr. W. Waugh, to have the patient wear a broad rubber band at the base of the penis close to the pubes over the dorsal vein during sexual intercourse, in order to prevent too rapid return of the venous blood. If it is found that he can thus obtain a good full erection, it is evident that too much venous blood is being carried off.

After having made the diagnosis the treatment is very simple. An elastic ligature is placed around the base of the penis, as near the pubes as possible, and a hypodermatic injection of ¼ grain cocaine is made by the side of the vein for local anesthesia. The vein is then cut down upon, grasped with forceps, ligated at its proximal and distal ends and divided between the ligatures. Heavy catgut should be used for ligatures, as they are readily absorbed and will cause no inconvenience to the patient. I first used small silk ligatures, but found that they would slough out instead of becoming incorpórated, owing to the physiological action of the organ. Furthermore, if silk were used and allowed to become encapsulated, it would act as a constant irritant. An iodoform collodion dressing is then applied and is allowed to remain for three days, when it is removed and the patient discharged, but instructed not to have coitus for two weeks.

Dr. Barthlow was the first to call attention to impotency due to a too rapid emptying of blood from the penis, and advocated for its treatment the hypodermatic injection of ergotin along the side of the dorsal vein. I have employed this procedure many times without any good results, as the injection causes a great deal of irritation, pain, swelling and as the use of ergot is almost always followed by faintness.

In 1893 Dr. W. Waugh reported a case of impotency in which he ligated the dorsal vein with excellent results. Following Dr. Waugh's suggestion I have performed this operation three times.

CASE 1.—G. T., aged twenty-three, came to me and stated that since an attack of gonorrhea three years before he had never been able to have a free erection, and although he had strong sexual desire he was unable to accomplish coitus. Upon examination a stricture was found in the pendulous portion of the urethra, with a prostatorrhea. The stricture was dilated until the urethra was restored to its normal caliber, and the prostatorrhea treated until cured. Still he remained impotent. I then noticed there was an enlargement of the dorsal vein of the penis. Injections of ergotin were made along the side of the vein without any results. Later on I ligated the dorsal vein of the penis, which immediately filled with blood. This patient is now married, and reports that since the operation he has had intercourse regularly without a single failure.

CASE 2.—S. J. R., aged twenty-seven, consulted me for loss of erectile power, which condition had been coming on gradually for the last few months. He gave the history of having had gonorrhea six years before, since which time he always noticed a slight discharge from the urethra. Upon examination I fonnd a chronic prostatorrhea, which at the time I considered to be the cause of the impotency. This condition was treated and cured without benefiting the impotency. Various tonics were also administered without any benefit. As I noticed that the dorsal vein was somewhat enlarged I performed ligation, and since the operation the patient has never had any trouble.

CASE 3.—Mr. L. consulted me for impotency. Family and personal history good; he practiced masturbation when a boy. Upon examination I found an enlargement of the dorsal vein, otherwise the genital organs were in perfect condition. I ligated the dorsal vein of the penis, and he now reports himself "as good as any man."

In a late article on this subject by Dr. J. A. Murry, he reports five cases, in all of which he had good results from this operation.

Dr. J. P. Lynch also reports a case in which, after having first operated for varicocele, with the expectations of curing the impotency, without result, he ligated the dorsal vein with excellent results.

CONCLUSION.—1. Ligation of the dorsal vein of the penis will cure impotency in those cases in which there is a too rapid emptying of blood from the penis, collateral circulation being carried on through the prostatic plexus.

2. Statistics show that this operation corrects the abnormal circulation in the penis.—*Med. Standard.*

1003 Columbus Memorial Building.

